# The efficacy and cost effectiveness of a multidisciplinary intervention strategy for the treatment of benign joint hypermobility syndrome (BJHS) in childhood. a randomised, single centre parallel group trial. (The bendy study)

**DOI:** 10.1186/1546-0096-12-S1-P58

**Published:** 2014-09-17

**Authors:** Peter J Bale, Vicky Easton, Holly Bacon, Emma Jerman, Kate Armon

**Affiliations:** 1 Norwich Medical School, University of East Anglia, UK; 2 Paediatric Physiotherapy, Norfolk and Norwich University Hospital, UK; 3 Occupational therapy, University of East Anglia, UK; 4 Jenny Lind Children's Department, Norfolk and Norwich University Hospital, Norwich, UK

## Introduction

Joint hypermobility is common in childhood and can be associated with musculoskeletal pain and dysfunction. Current management is delivered by a multidisciplinary team but evidence of efficacy is limited.

## Objectives

This clinical trial aimed to determine whether a structured multidisciplinary intervention resulted in improved clinical outcomes compared with standard care

## Methods

A prospective randomised, single centre parallel group trial comparing an 8-week individualised multidisciplinary intervention programme with current standard management (advice and a physiotherapy appointment). Children and young people (CYP) were assessed for pain, function, coordination and strength at baseline, 3 and 12 months.

## Results

119 CYP, aged 5 to 16 years, with symptomatic hypermobility were randomised to receive targeted multidisciplinary intervention (I) (n=59) or standard management (S) (n=60). Of these, 105 were followed to 12-months. There was a significant improvement in child and parent reported pain, coordination and strength. However, no added benefit could be shown from the intervention (Table 1). The number of CYP showing significant pain reduction (>=40%) was 27 (50.0%) (I) vs 21 (41.1%) (S). Those pain free at 12 months were 29 (56.9%) (I) vs 20 (45.5%) (S). The response was independent of the degree of hypermobility.

**Table 1:** 

	Baseline score (SD)	Rate of change over 12 months (95% CI)			
		
Outcome variable		Intervention group		Control	
Child pain assessment (0-5, zero is the best), n=103	2.31 (1.55)	-1.42	(-1.78 to -1.06)	-1.31	(-1.75 to -0.85)

Parent observed pain assessment (0-100 VAS, zero is the best) n=105	35.90 (26.46)	-6.09	(12.90 to 0.73)	-6.22	(-13.62 to 1.18)

Child health assessment questionanire (CHAQ) (0-3, zero is the best), n=104	0.82 (0.63)	+0.02	(-0.12 to 0.16)	-0.03	(-0.13 to 0.64)

Child health 9 dimensional utility (CHU9D) (0-1, zero is the worst), n=104	0.85 (0.11)	+0.02	(-0.01 to 0.04)	+0.002	(-0.02 to 0.03)

Movement assessment battery for children (M-ABC) (0-100, zero is theworst), n =104	34.56 (28.61)	+2.60	(-2.92 to 8.11)	+8.51	(3.17 to 13.86)

Grip Strength (Dynamometer), n=104	57.29 (28.30)	+4.55	(0.16 to 8.94)	+6.75	(2.85 to 10.66)

## Conclusion

This is the first RCT to compare a structured multidisciplinary intervention with standard care in symptomatic childhood hypermobility. The study demonstrates significant improvement among subjects but no additional benefit from targeted intervention. The findings emphasise the benefit of information and physiotherapy, but highlight the difficulty in demonstrating subtle benefit from specific interventions without better tools for case definition and outcomes assessment.

## Trial registration identifying number

UKCRN Portfolio 9366.

## Disclosure of interest

None declared.

